# Ultralow‐Power Machine Vision with Self‐Powered Sensor Reservoir

**DOI:** 10.1002/advs.202106092

**Published:** 2022-03-13

**Authors:** Jie Lao, Mengge Yan, Bobo Tian, Chunli Jiang, Chunhua Luo, Zhuozhuang Xie, Qiuxiang Zhu, Zhiqiang Bao, Ni Zhong, Xiaodong Tang, Linfeng Sun, Guangjian Wu, Jianlu Wang, Hui Peng, Junhao Chu, Chungang Duan

**Affiliations:** ^1^ Key Laboratory of Polar Materials and Devices (MOE) Ministry of Education Department of Electronics East China Normal University Shanghai 200241 China; ^2^ Zhejiang Lab Hangzhou 310000 China; ^3^ Collaborative Innovation Center of Extreme Optics Shanxi University Shanxi 030006 China; ^4^ Centre for Quantum Physics Key Laboratory of Advanced Optoelectronic Quantum Architecture and Measurement (MOE) School of Physics Beijing Institute of Technology Beijing 100081 China; ^5^ Institute of Optoelectronics Frontier Institute of Chip and System Fudan University 220 Handan Road Shanghai 200433 China; ^6^ Institute of Optoelectronics Fudan University 220 Handan Road Shanghai 200433 China

**Keywords:** Cs_2_AgBiBr_6_, in‐sensors, lead‐free double perovskites, machine vision, reservoir

## Abstract

A neuromorphic visual system integrating optoelectronic synapses to perform the in‐sensor computing is triggering a revolution due to the reduction of latency and energy consumption. Here it is demonstrated that the dwell time of photon‐generated carriers in the space‐charge region can be effectively extended by embedding a potential well on the shoulder of Schottky energy barrier. It permits the nonlinear interaction of photocurrents stimulated by spatiotemporal optical signals, which is necessary for in‐sensor reservoir computing (RC). The machine vision with the sensor reservoir constituted by designed self‐powered Au/P(VDF‐TrFE)/Cs_2_AgBiBr_6_/ITO devices is competent for both static and dynamic vision tasks. It shows an accuracy of 99.97% for face classification and 100% for dynamic vehicle flow recognition. The in‐sensor RC system takes advantage of near‐zero energy consumption in the reservoir, resulting in decades‐time lower training costs than a conventional neural network. This work paves the way for ultralow‐power machine vision using photonic devices.

## Graphical abstract

In this work, photovoltaic devices are innovatively used as self‐powered reservoirs. Ultralow‐power machine vision is achieved by the self‐powered sensor reservoir with Cs_2_AgBiBr_6_ photonic devices. Both image processing and dynamic video analysis are energy‐efficiently achieved in this in‐sensor reservoir computing system. The accuracy is 99.67% for face classification and 100% for dynamic vehicle flow recognition.

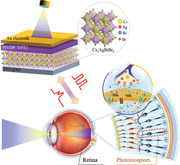

## 1 Introduction

**Figure 1:**
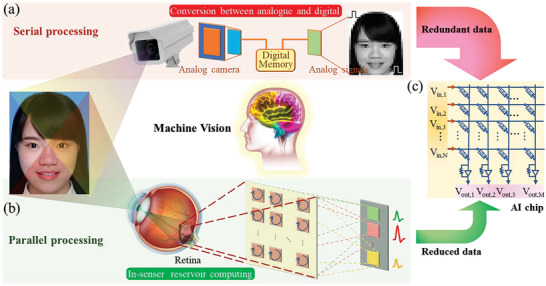
Schematics of machine vision systems. a) A traditional machine vision system where visual information is captured by an analog camera, converted into digital/analog signals for memory unit/afterward in‐memory AI chip. b) A bionic visual system by an in‐sensor RC. The full‐analog processing and reduced data movement result in a low latency and high energy efficiency. c) The in‐memory AI chip with a high‐density memristor crossbar array.

Machine vision mainly conducts heavy matrix multiplication for image‐processing tasks, such as pattern recognition and object detection.^[^ ^1^ ^]^ In a traditional machine vision system, visual information is captured by an analog camera and converted into digital signals for memory units and afterward computing units.^[^ ^2^, ^3^ ^]^ The separation of processor and memory in the traditional computing unit based on a von Neumann architecture causes huge latency and energy consumption during the matrix‐multiplication processing,^[^ ^4^, ^5^, ^6^ ^]^ which hinders the delay‐sensitive applications such as driverless vehicles, robotics, or industrial manufacturing. Replacing the von Neumann architecture with an in‐memory computing technology is the most convenient strategy.^[^ ^7^, ^8^ ^]^ But it suffers additional digital‐to‐analog conversion (DAC) due to the analog processing in the In‐memory artificial intelligence (AI) chip (**Figure** **1**a,c). Furthermore, the honest transmission of redundant data from camera causes high power consumption.

It gives a better solution in human visual system. The sensory neurons detect analog optical signals and perform a first‐stage image processing in which the amount of data is greatly reduced before the following visual signal processing in visual cortex of the human brain.^[^ ^9^ ^]^ The full‐analog processing and reduced data movement result in a low latency and high energy efficiency (Figure 1b). A neuromorphic visual system integrating optoelectronic synaptic devices to form the in‐sensor computing is newly proposed^[^ ^9^, ^10^ ^]^ and triggers much attention.^[^ ^1^, ^2^, ^3^, ^11^, ^12^, ^13^, ^14^, ^15^, ^16^ ^]^ It is experimentally demonstrated that the visual pre‐processing by a photoconductive synaptic device array can improve the processing efficiency and image recognition rate.^[^ ^1^, ^9^, ^12^, ^14^ ^]^ Mennel et al. show that both supervised and unsupervised learning algorithms can be performed in photovoltaic sensors with an ultrahigh throughput of 20 million bins per second.^[^ ^3^ ^]^ Very recently, Sun et al. reported that the reservoir algorithm can also be achieved based on photoconductive sensors.^[^ ^17^ ^]^ Thus, the reservoir computing (RC) opens a way for high energy‐efficient machine vision because it can efficiently process complex and temporal data with low‐training cost, taking advantage of that only the readout function needs to be trained.^[^ ^18^, ^19^, ^20^, ^21^ ^]^ Among these optoelectronic synapses, the photocurrent other than photoconductance is preferred to simulate the synaptic weight because the photovoltaic work mode makes the photocurrent device free of energy consumption. Aiming the high energy efficiency, using self‐powered photovoltaic devices to constitute the in‐sensor RC is extremely appealing, but not realized yet due to the transient characteristic of photocurrents.

In this work, an ultralow‐power machine vision is implemented based on the in‐sensor RC constituted by self‐powered Au/P(VDF‐TrFE)/Cs_2_AgBiBr_6_/ITO devices. The designed band alignment with a potential well on the shoulder of Schottky energy barrier greatly extends the dwell time of photon‐generated carriers in the space‐charge region, permitting the nonlinear interaction of photocurrents stimulated by spatiotemporal optical signals. This mechanism is unambiguously confirmed by the polarization‐modulated coupling strength between optoelectronic responses. We demonstrate both image processing, such as face classification, and dynamic video analysis, such as vehicle flow detection, can be energy‐efficiently performed by the photocurrent‐encoded in‐sensor RC. It shows an accuracy of 99.97% to classify face images with decades‐lower training cost than conventional neural networks and an accuracy of 100% to classify orientation of dynamic vehicle flow.

## 2 Results and Discussion

Organic‐inorganic hybrid halide perovskites (OHPs, CH_3_NH_3_PbX_3_) are widely used in optoelectronic devices due to its excellent optoelectronic properties. However, the intrinsic instability and environmental unfriendliness of OHPs greatly limit their further commercial application.^[^ ^22^, ^23^ ^]^ All‐inorganic lead‐free double perovskites, especially Cs_2_AgBiBr_6_, have become one of the most promising alternatives to OHPs for optoelectronics^[^ ^24^ ^]^ due to their advantages of environmental stability, low toxicity, and simple solution‐processing method,^[^ ^25^, ^26^ ^]^ allowing for applications requiring large area, low cost or mechanical flexibility. In addition, ferroelectric materials are a kind of functional materials with tunable spontaneous polarization by external electric field^[^ ^27^, ^28^ ^]^ and are always adopted in synaptic transistors as gate insulators to control the conductance variations.^[^ ^29^, ^30^, ^31^ ^]^

### 2.1 Device Structure

**Figure 2:**
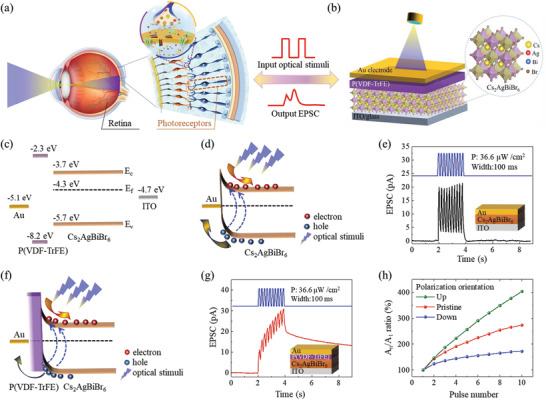
Tunable EPSC in Cs_2_AgBiBr_6_ photonic synapses. a) Schematic diagrams of human visual system. b) The diagram of the designed photonic synapse. Inset shows the crystal structure of Cs_2_AgBiBr_6._ c) The band energy alignment of Au, P(VDF‐TrFE), Cs_2_AgBiBr_6_, and ITO. d) The band diagram of the Au/Cs_2_AgBiBr_6_/ITO device under optical stimulus. e) EPSC triggered by 10 optical pulses on the Au/Cs_2_AgBiBr_6_/ITO device. f) The band diagram of the Au/P(VDF‐TrFE)/Cs_2_AgBiBr_6_/ITO device under optical stimulus. g) EPSC triggered by 10 optical pulses on the Au/P(VDF‐TrFE)/Cs_2_AgBiBr_6_/ITO device. h) The evolution of EPSC coupling for three polarization states that is up after negative pooling, random for the pristine film, and down after positive pooling, respectively.

The photonic synapse in this work is designed for directly receiving optical signals and giving spatiotemporal‐linked responses, which simulates the function of photoreceptors and following synapses in human visual system (**Figure** **2**a). A simple structure combining a photosensitive layer of Cs_2_AgBiBr_6_ and ferroelectric polymer layer was fabricated with top Au electrode and bottom ITO glass electrode to form the bionic photonic synapse (Figure 2b). The Cs_2_AgBiBr_6_/P(VDF‐TrFE) heterostructure with clear interface and undamaged phase was confirmed by using scanning electron microscopy (SEM) (Figure S1, Supporting Information) and X‐ray diffraction (XRD) spectroscopy (Figure S2, Supporting Information). The UV–vis absorption spectra show that Cs_2_AgBiBr_6_ has an absorption peak around 445 nm, and the additional layer of P(VDF‐TrFE) doesn't affect its absorption characteristic (Figure S3, Supporting Information). Thus, optical pulses with a wavelength of 445 nm were used to testify the device functions.

### 2.2 Tunable Photocurrents in Cs_2_AgBiBr_6_ Photonic Synapses

The ferroelectric polymer layer plays a crucial role in the modulation of optoelectronic response in the Cs_2_AgBiBr_6_ photonic synapse. The band energy alignment of Au, P(VDF‐TrFE), Cs_2_AgBiBr_6_, and ITO materials obtained from literatures^[^ ^32^, ^33^ ^]^ are demonstrated in Figure 2c. The work function of 5.1 eV in Au electrode and 4.7 eV in ITO electrode facilitate the Schottky contact at Au/n‐type Cs_2_AgBiBr_6_ interface and almost ohmic contact at n‐type Cs_2_AgBiBr_6_/ITO interface. Without the P(VDF‐TrFE) layer, a direct Schottky barrier at the Au side (Figure 2d) permits photocurrent through the device when it is under optical pulses, and the photocurrent fade away rapidly after removing the light pulses (Figure 2e). This photocurrent represents excitatory post‐synaptic current (EPSC) as we treat our device as a synapse. The EPSC in the Au/Cs_2_AgBiBr_6_/ITO device originates from the separation of photon‐generated carriers by the built‐in electric field in the Schottky barrier. When the source of photon‐generated carriers is shut down with the turning off of optical pulse, the built‐in electric field could remove these residual carriers away and the EPSC disappears. There is slight coupling between ESPC under ten successive optical pulses with a width of 100 ms, power density of 36.6 µW cm^−2^, and an internal span of 100 ms (Figure 2e). When a P(VDF‐TrFE) layer is introduced between the Au electrode and Cs_2_AgBiBr_6_ layer, the high binding energy of the valence electron states in P(VDF‐TrFE) layer gives an energy potential well for hole carriers at the P(VDF‐TrFE)/Cs_2_AgBiBr_6_ interface (Figure 2f). This energy potential well makes the photon‐generated hole carriers difficult to migrate to the Au electrode, which weakens the EPSC under the optical pulse, but much extends the lifetime of EPSC after removing the optical stimulus. Furthermore, a strong coupling between EPSC is observed and the amplitude of EPSC continually strengthened under the ten successive optical pulses (Figure 2g). The EPSC coupling can be quantified by the facilitation factor (A_n_/A_1_), where A_n_ is the amplitude of EPSC under the *n*th optical pulse. The amplitude of EPSC and their coupling strength can be tuned by altering the ferroelectric polarization orientation through modulating the shape of the energy potential well (Figures S4 and S5, Supporting Information). The EPSC coupling for three polarization states, that is up after negative pooling, random for the pristine film, and down after positive pooling, are presented in Figure 2h, from which one can see that the polarization reversal induces a variation of 234.56% in the EPSC coupling. It is noted that the positive relationship between the coupling strength of optoelectronic response and the depth of energy potential well which is modulated by polarization further confirms the validity of the mechanism (Note S2, Supporting Information). The tunable EPSC enables Cs_2_AgBiBr_6_ photonic synapse potential for the rapid In‐sensor computing.^[^ ^2^, ^3^ ^]^

### 2.3 The Nonlinear and Spatiotemporal‐Linked EPSC and Device Variation

Besides the tunability, the long‐lifetime EPSC itself and their couplings are also appealing for optical‐involved information processing. The EPSC characteristics in Cs_2_AgBiBr_6_ photonic synapse are further explored. Under the stimulus of an optical pulse with a width of 100 ms and power density of 36.6 µW cm^−2^, the EPSC instantly reaches to ≈10.6 pA and then decreases gradually after removing the stimulus (Figure S6, Supporting Information). The amplitude of EPSC can be modulated by the width (Figure S7a,b, Supporting Information) and power density (Figure S7c,d, Supporting Information) of the optical pulses. With a constant power density of 36.6 µW cm^−2^, as the pulse width increases from 0.1 to 4.5 s, the EPSC increases from 10.9 to 145.3 pA, achieving nonlinear 1233% enhancement (Figure S7b, Supporting Information). With a constant pulse width of 100 ms, the EPSC increased 524% (from 10.1 to 63.1 pA) as the power density increased from 36.6 to 383.4 µW cm^−2^ (Figure S7d, Supporting Information).

**Figure 3:**
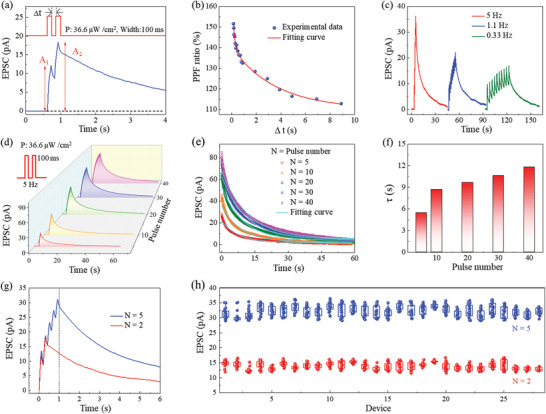
The nonlinear and spatiotemporal‐linked EPSC and device variation. a) PPF behavior stimulated by a pair of optical pulses with the time interval (Δt) of 100 ms. b) Dependence of the PPF ratio (defined as A_2_/A_1_ × 100%) on the time interval (Δt). c) SRDP triggered by different rates of optical pulse. d) SNDP triggered by different numbers of optical pulses. e,f) Decay curves and corresponding decay time (*τ*) of SNDP in Figure 3d. g,h) The uniformity and robustness were investigated by evaluating the EPSC response from 28 random‐picked devices stimulated by 2 optical pulses and 5 optical pulses. All measurements were performed at 0 V bias.

Pair‐pulse facilitation (PPF) is one of the most representative behaviors of biological synapses. When two optical pulses are applied to the device sequentially, the EPSC value of the second optical pulse is higher than the first one because of the EPSC coupling. **Figure** **3**a presents the PPF behavior stimulated by two optical pulses with a width of 100 ms, power density of 36.6 µW cm^−2^, and Δt of 100 ms. The PPF ratio can be modulated by the interval time (Δt) between the pair of optical pulses. As shown in Figure 3b, the PPF ratio (defined as A_2_/A_1_ × 100%) decreases from 152% to 112% as the Δt increases from 150 ms to 9 s. The experimental data (blue dots) could be well fitted by the double exponential decay function:^[^ ^34^ ^]^

(1) $$y\;=\;1+C_{1}\;\text{exp}\left(-\Delta t/\tau_{1}\right)+C_{2}\;\text{exp}\left(-\Delta t/\tau_{2}\right)\;$$

where *τ*_1_ = 0.16 s and *τ*_2_ = 3.57 s are the characteristic relaxation time and *C* is the initial facilitation magnitude. Correspondingly, temporal plasticity such as spiking‐rate dependent plasticity (SRDP) and spike‐number dependent plasticity (SNDP) can be implemented by using our synaptic device. Figure 3c shows the EPSC triggered by 10 optical pulses with different frequencies of 5, 1.1, and 0.33 Hz, respectively. The EPSC nonlinearly depends on the spiking rate, and the higher rate triggers the larger EPSC. Similar nonlinear EPSC response with a spiking number is also observed (Figure 3d). The EPSC decay curves of SNDP (Figure 3e) can be well fitted by an exponential decay function^[^ ^35^ ^]^

(2) $$y\;=y_{0}+\exp[-\left(t/\tau )\right]\;$$

where *τ* is the characteristic relaxation time of the decay process. Figure 3f shows the decay time (*τ*) increases from 5.44 to 11.77 s as the optical spiking number increases from 5 to 40. These nonlinear, spatiotemporal‐linked, and short‐term EPSC responses to either spiking width, power density, rate, or spiking number, make the device potential to process complex temporal information.

Before the application demonstration, the uniformity and robustness of our Cs_2_AgBiBr_6_ photonic synapses were investigated by evaluating the EPSC responses of 28 random‐picked devices stimulated by 2 and 5 optical pulses, respectively, and each device was repeatedly inspected for 20 times. The amplitude of EPSC at 1 s (Figure 3g) was taken for statistical analyses. As demonstrated in Figure 3h, all 28 devices show good uniformity and robustness. The typical cycle‐to‐cycle variation is 0.078 for 2 stimuli and 0.075 for 5 stimuli, and the device‐to‐device variation is 0.221 for 2 stimuli and 0.159 for 5 stimuli. The decrease of variation for either cycle to cycle or device to device may attribute to the canceling effect of superimposed random variations.

### 2.4 In‐Sensor Reservoir Computing for Image Classification

RC is a simple and efficient brain‐like algorithm suitable for processing timing signals. In an RC system, a dynamic “reservoir” of synapses with short‐term memory maps complex timing signals into a new space, represented by the state of the node in the reservoir. Because only the weight of the output connection between the reservoir and the output layer needs to be trained, RC greatly reduces the training cost of the network. The necessary short‐term memory in RC can be simulated by the nonlinear and spatiotemporal‐linked EPSC responses that are triggered directly by optical signals. Thus, it is possible to build an In‐sensor RC system with Cs_2_AgBiBr_6_ photonic synapses.

**Figure 4:**
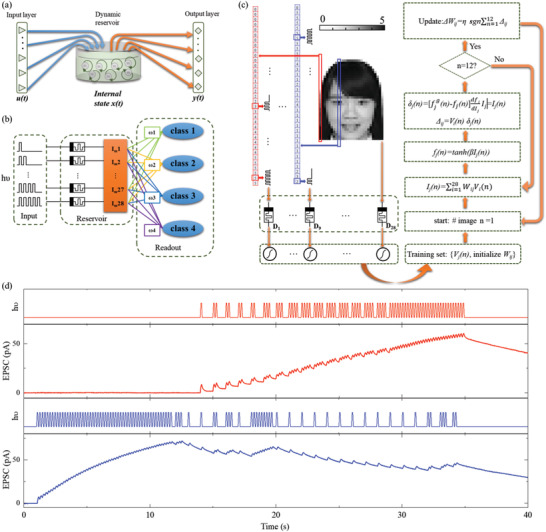
In‐sensor RC using Cs_2_AgBiBr_6_ photonic synapses. a) Schematic of a conceptual RC system, including input layer, dynamic reservoir, and output layer. b) Schematic of the RC system with optical pulse sequences as the inputs, the memristor reservoir, and a readout network. c) A concrete example for the process of face classification. d) EPSC evolutions of reservoir device one (D_1_) and device nine (D_9_) in Figure 4c.

**Figure** **4**a shows a conceptual RC system, including input layer, dynamic reservoir, and output layer. The transient input of all synapses in reservoir at time *t* forms the input state *u*(*t*). With the dynamic evolutions of reservoir internal state *x(t)*, the input *u*(*t*) can be successfully mapped to the high‐dimensional feature space as *y*(*t*). Then the high‐dimensional feature space *y*(*t*) is used as new inputs to the following network for processing. By encoding the space information to time‐series *u*(*t*), the network size for image processing is greatly reduced. For example, to classify gray‐scale face images with 28 columns × 35 rows pixels. A conventional neural network will cost 28 × 35 × n weights to recognize the topic one from n candidates. On the contrary, the RC system only needs 28 × n weights to implement the same recognition task by encoding 35 pixels in each column as time‐series *u*(*t*). Here 28 devices were selected to build the reservoir to map four gray‐scale face images (28 × 35 pixels) and a following 28 × 4 memristor network is simulated to accomplish the classification task.

**Figure 5:**
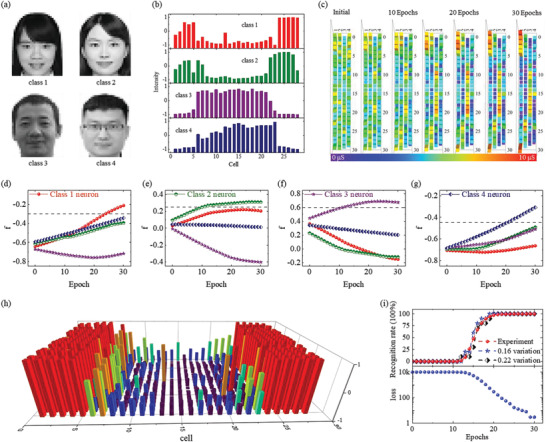
Face image classification by the In‐sensor RC system. a) Four ID photos were chosen for face recognition and preloaded to 980 pixels in 28 × 35 size. b) One group of normalized *y*(*t*) for four ID photos (class 1, class 2, class 3, and class 4). c) Weights of memristors in 28 × 4 array are updated during each training epoch. d–g) The readout function (*f*) value of the corresponding targeted neuron. h) 20 experimental *y*(*t*) spaces of class 1. i) Top panel: the recognition rate for classification of class 1. Both experimental *y*(*t*) spaces and simulated *y*(*t*) spaces with a random variation of 0.16 and 0.22 are involved to obtain the recognition rate. Bottom panel: The failed number for classification of class 1 in 10 240 tests.

Figure 4b illustrates the schematic of the RC system, consisting of input, reservoir, and readout. The optical pulse sequences are fed to the reservoir as inputs, which will cause a change in the EPSC values in the devices in the reservoir. A concrete example is shown in Figure 4c to explain the process of face classification. Four identification photos (ID photo) from East China Normal University are chosen and preloaded to 28 × 35 pixels size (**Figure** **5**a). These ID photos are in grayscale and each pixel value ranges from 0 to 5 to reflect their gray level where the pure white color corresponds to 0 and pure dark color to 5, respectively. Pixels in the same column are serially encoded to one optical pulse sequence where the pixel value matches the optical pulse number.

In our devices, the EPSC decay curves of SRDP can be well fitted by the exponential decay function of Equation (2) (Figure S8, Supporting Information). Figure S8c, Supporting Information, shows the decay time (*τ*) increases from 11 s to 21.73 s as the spiking frequency of ten optical pulses increases from 0.2 to 8.33. The SRDP ratio (defined as A_10_/A_1_) increases from 130% to 301% with the pulse frequency increasing from 0.1 to 5.55 Hz and approaching saturation at 5 Hz (Figure S8d, Supporting Information). Thus, to fully utilize the coupling between stimulus, a frequency of 5 Hz (width of 100 ms and interval of 100 ms) are used to determine the gray level of each pixel. Considering the decay time of tens of seconds, a frequency of 1 Hz (1 s for each pixel) is used to handle these images with 35 pixels in each column. Thus, each pixel costs a period of 1 s in which each optical pulse survives 0.1 s followed by a cease span of 0.1 s. For example, as demonstrated in Figure 4c,d, there are no optical pulses during the period of 1 s if the pixel value is 0. There are three optical pulses at the first 0.6 s if the pixel value is 3, and there are five optical pulses during the whole 1 s if the pixel value is 5. All 28 optical pulse sequences are input to the 28 reservoir devices and the EPSC responses are recorded. Figure 4d shows EPSC evolutions of reservoir device one (D_1_) and device nine (D_9_) in Figure 4c. Because of the nonlinear EPSC coupling, different optical pulse sequences trigger unequable EPSC responses. The amplitude of EPSC at 35 s is collected from the 28 reservoir devices as the new feature space *y*(*t*). An activation function is used to further differentiate the intensity distribution in *y*(*t*) and converts *y*(*t*) to normalized voltage inputs for the following network (Note S4, Supporting Information).

Through our in‐sensor reservoir system, the 28 × 35 pixels information in each photo are abstracted to a new feature *y*(*t*) space with only 28 elements. One group of normalized *y*(*t*) for four ID photos (class 1, class 2, class 3, and class 4 in Figure 5a are shown in Figure 5b. Note that the abstraction from *u*(*t*) to *y*(*t*) is not a rigorous one‐two‐one process since it cannot be guaranteed that all 5^35^ kinds of optical pulse sequence triggers distinguishable EPSC responses. To test the function of our in‐sensor reservoir system, a virtual 28 × 4 memristor network is trained and used for the face classification task. Following the flow chart in the right panel of Figure 4c, the training simulation is performed using the new *y*(*t*) inputs and a Manhattan update rule.^[^ ^36^, ^37^ ^]^ Ten groups of experimentally collected *y*(*t*) inputs for class 1 are used to train the readout memristor network. All weights of memristors in 28 × 4 arrays are initially randomly selected with a conductance range between 3 and 7 µS. Then all weights are updated during each training epoch (Figure 5c). Correspondingly, the value of readout function (*f*) of the targeted neuron continuously increases as the training epoch and exceeds others after 20 epochs (Figure 5d–g). To check the accuracy, 10 240 *y*(*t*) spaces are created by randomly picking experimental *y*_i_(t) that is not used during the training process, that is, from the other ten groups of experimentally collected *y*(*t*) inputs in Figure 5h. After each training epoch, all 10 240 *y*(*t*) spaces and simulated *y*(*t*) spaces with a random variation of 0.16 and 0.22 are inputted to the 28 × 4 memristor network to test the recognition rate. The results show that the in‐sensor reservoir system based on Cs_2_AgBiBr_6_ photonic synapses can achieve a satisfactory recognition rate of 99.97% for face classification which accords well with the simulation results with the variation of 0.16 and 0.22 (Figure 5i).

We have performed another training simulation on a readout memristor network with uniform initial weights. All weights of memristors in 28 × 4 arrays are initially programmed to the same conductance of 5 µS and updated during each training epoch (Figure S9e, Supporting Information). Interestingly, the value of readout function (f) of the targeted neuron equals to others at the initial, exceeds others after the first epoch, and continuously increases as the training epoch evolves (Figure S9a–d, Supporting Information). After each training epoch, all 10 240 *y*(*t*) spaces same as that in the case of random initial weights and simulated *y*(*t*) spaces with a random variation of 0.16 and 0.22 are inputted to the 28 × 4 memristor network to test the recognition rate. The results show that the in‐sensor reservoir system can achieve a satisfactory recognition rate of 99.67% for face classification (Figure S9f, Supporting Information), which approaches the result of 99.97% for the readout memristor network with random initial weights. These results imply that the accuracy is slightly dependent on the initial weights of the readout memristor network.

### 2.5 In‐Sensor Reservoir Computing for Detecting Vehicle Flow

**Figure 6:**
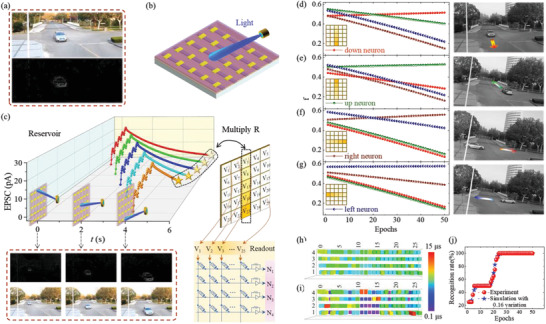
In‐sensor RC for detecting vehicle flow. a) Optical (top panel) and DSV (bottom panel) image at a crossroad. b) The sensor reservoir constituted by an array of 5 × 5 Cs_2_AgBiBr_6_ photonic synapses, in which the moving vehicle can be detected. c) A concrete example of the reservoir array responding to the up‐to‐down vehicle flow. The new *y*(*t*) spaces are inputted to the following 25 × 4 memristor array for classification. d–g) The readout function (*f*) value of the corresponding targeted neuron that connects to the outputs of the 25 × 4 memristor array. h,i) The weights of memristors in the 25 × 4 array before and after the 50 training epochs. j) The recognition rate for detecting vehicle flow. Both experimental *y*(*t*) spaces and simulated *y*(*t*) spaces with random variation of 0.16 are involved to obtain the recognition rate.

By directly collecting the spatiotemporal optoelectronic signals as *u*(*t*), the In‐sensor RC is suitable for processing dynamic video information. A scene of road camera with in‐sensor RC for monitoring vehicle flow is designed. As demonstrated in **Figure** **6**a,b, an array of 5 × 5 Cs_2_AgBiBr_6_ photonic synapses forms the sensor reservoir. The spatial vehicle information is optically inputted to the sensor reservoir by synchronizing vehicle pixels from the dynamic vision sensor (DSV) video (bottom panel in Figure 6a). Figure 6c shows how the reservoir array responds to the up‐to‐down vehicle flow. During the vehicle movement, all 25 Cs_2_AgBiBr_6_ photonic synapses are active and release EPSC once they receive the five optical stimuli that symbolize the position of vehicle pixels. Vehicle flows with different orientations give disparate spatiotemporal EPSC responses, resulting in distinguishable EPSC pattern.

Following the flow chart in the right panel of Figure 4c, a virtual 25 × 4 memristor network is trained and used for the vehicle flow recognition task. Weights of memristors in 25 × 4 arrays are randomly programmed around conductance of 5 µS (Figure 6h) and updated during each training epoch. During the training process, the supervised *f* value continuously increases for the targeted *y*(*t*) inputs and decreases for other unintended *y*(*t*) inputs. After 50 times training epoch, the new‐arranged conductance weights array (Figure 6i) gives the maximum *f* value for each orientation neuron when the vehicle flows with the same orientation are inputted (Figure 6d–j). 20 vehicle flows with down orientation are experimentally inputted to this In‐sensor RC system after each training epoch to evaluate the accuracy. As shown in Figure 6j, a pleasant recognition rate of 100% after 24 training epochs are obtained, which is comparable with the simulation results with a variation of 0.16.

### 2.6 Stability and Energy Consumption

Cs_2_AgBiBr_6_ is one of the most promising alternatives to OHPs for optoelectronic devices due to their advantages of environmental stability. The stability of the Cs_2_AgBiBr_6_‐based photonic device was investigated by keeping in an ambient environment (the relative humidity was about 60% and the temperature was around 25 °C) for 8 weeks. Figure S10a, Supporting Information, shows the XRD patterns of Cs_2_AgBiBr_6_/P(VDF‐TrFE) hybrid film before and after eight week's storage, and no obvious changes were observed. The response to single optical pulse (power density and pulse width were 28.3 µW cm^−2^ and 100 ms, respectively) was almost the same with after 56 days’ storage, as illustrated in Figure S10b, Supporting Information (Inset was the EPSC triggered by the same input from day 1 to day 56). The results further confirm the good reproducibility and environmental stability of the Cs_2_AgBiBr_6_‐based photonic device.

A significant advantage of using the RC system is the reduction of network size and training cost. For example, in our in‐sensor RC system, the spatial information can be encoded to temporal signals so only a small network needs to be trained. For example, the 28 × 35 pixels information of ID photo in this work are abstracted to new feature *y*(*t*) space with only 28 elements. The following memristor network has 28 inputs for the 28 *y*(*t*) elements and 4 outputs for classifying four ID photos, 28 × 4 weights need to be trained. However, a conventional neural network will have 28 × 35 inputs for 28 × 35 pixels and 4 outputs for classifying four ID photos, 28 × 35 × 4 weights need to be trained.^[^ ^36^, ^37^ ^]^ On the bias of hardware size, both the energy consumption and training cost are decades time reduced. This saved energy value will increase very quickly if one or more hidden layers are involved.

Compared with other conductance‐based reservoir layers, the photovoltaic reservoir devices in this work not only avoid both the analog‐to‐digital conversion (ADC), from an analog camera to memory unit, and the DAC, from memory unit to the neural network hardware, but also free of energy consumption in itself. The energy consumption of per synaptic event can be calculated by *V* × *I* × *t*, where *V* is the measuring voltage, *I* is the EPSC of the device, and *t* is the optical pulse duration. Near‐zero energy is in principle consumed in our two‐terminal device, due to its voltage‐free photovoltaic (i.e., self‐powered) work mode. The comparation of energy cost per reservoir operation in the reservoir layer between reported reservoir systems is summarized in **Table** **1**. It demonstrates that our reservoir layer takes advantage of near‐zero energy consumption. The energy is consumed only in the following processes, such as current‐to‐voltage conversion and weights programming in the readout memristor network. Note that, when it processes spatial information, the spatial information is converted to temporal information by the reservoir layer to save the following hardware size,^[^ ^1^ ^]^ which is time‐consuming. The reservoir system is very suitable for directly processing temporal information that has a similar time scale, such as trajectory analysis for car, steamer, or animals in our reservoir devices with a time scale of seconds.

**Table 1:** Comparation of energy cost per reservoir operation in the reservoir layer between reported reservoir systems

Device unit	mechanism	Classifier task	Energy cost per reservoir operation	Ref.
W/WO_x_/Pd	Resistive synapse	Digit classification	≈ 1 pJ	^[^ ^18^, ^20^ ^]^
Pt/Ag‐doped SiO_2_/Pd	Diffusive synapse	Digit classification	≈ 1 nJ	^[^ ^19^ ^]^
Au/Cr/SnS/Cr/Au	Optoelectronic synapse	Language classification	≈ 5 nJ for electrical ≈ 100 nJ for optical	^[^ ^17^ ^]^
Au/P(VDF‐TrFE) /Cs_2_AgBiBr_6_/ITO	Photonic synapse	Face classification	0	This work

## 3 Conclusion

In this work, we experimentally demonstrate that robust nonlinear interaction of photocurrents responding to spatiotemporal optical signals can be achieved by embedding a potential well on the shoulder of Schottky energy barrier in Au/P(VDF‐TrFE)/Cs_2_AgBiBr_6_/ITO device. The tunable photocurrent by the polarization reversal through external electric field makes the Cs_2_AgBiBr_6_ photonic synapses appealing for conventional In‐sensor computing.^[^ ^3^ ^]^ Besides this, these nonlinear and spatiotemporal‐linked photocurrents triggered directly by optical signals are intently used to constitute an In‐sensor RC system. Both image processing (face classification) and dynamic video analysis (vehicle flow recognition) are energy‐efficiently achieved with high accuracy. Notably, near‐zero energy is in principle consumed in Cs_2_AgBiBr_6_ photonic synapses due to its voltage‐free photovoltaic work mode. Furthermore, by encoding the spatial information to temporal signals, only a much smaller network needs to be trained in the In‐sensor RC system. Thus, the proposed machine vision by self‐powered sensor RC based on Cs_2_AgBiBr_6_ photonic synapses takes significant advantage of low energy consumption.

## 4 Experimental Section

### Materials

Cesium bromide (CsBr, 99.9%), silver bromide (AgBr, 98%), P(VDF‐TrFE) (70:30), and diethyl carbonate (99%) were purchased from Aladdin Company. Bismuth bromide (BiBr_3_, 99%) was obtained from Alfa Aesar. Dimethylsulfoxide (DMSO, 99.9%) was sourced from J&K company.

### Preparation of Cs_2_AgBiBr_6_ Solution

CsBr (2 mmol, 426 mg), AgBr (1 mmol, 188 mg), and BiBr_3_ (1 mmol, 449 mg) were mixed in 2 mL DMSO to acquire 0.5 m Cs_2_AgBiBr_6_ precursor, and stirred at 70 °C for 2 h. A 0.22‐µm filter was used to filter the precursor solution to obtain a clear yellow solution.

### Device Fabrication

Indium‐tin oxide (ITO) glass was first cleaned by ultrasonication for 20 min with deionized water, acetone, alcohol, and isopropyl alcohol, respectively, and dried with nitrogen gas for further use. The Cs_2_AgBiBr_6_ film was synthesized by a simple one‐step spin‐coated method and treated by a typical low‐pressure‐assisted (LPA) method according to previous reports.^[^ ^38^, ^39^ ^]^ First of all, 60 µL 0.5 m Cs_2_AgBiBr_6_ solution was spin‐coated on the ultraviolet‐ozone treated ITO substrate at a low speed of 500 rpm for 5 s, followed by a high speed of 3000 rpm for 60 s to get a transparent yellow film. Then, the film was transferred to a low‐pressure chamber for 10 min. Next, the Cs_2_AgBiBr_6_ film was annealed at 280 °C for 5 min. After cooling down to the room temperature, 70 µL 2.5 wt.% P(VDF‐TrFE) (70:30) in diethyl carbonate was spin‐coated on the fabricated Cs_2_AgBiBr_6_ film at a low speed of 500 rpm for 5 s, followed by a high speed of 4000 rpm for 30 s. The sample was annealed at 140 °C for 4 h to enhance the crystallinity of the ferroelectric hybrid film. At last, about 50 nm Au was deposited by thermal evaporation to form the Au/P(VDF‐TrFE)/Cs_2_AgBiBr_6_/ITO artificial photonic synapse device. The Au top electrodes were fabricated under very “soft” deposition conditions to minimize the ferroelectric polymer film damage (background vacuum of ≈10^−4^ mbar and evaporation vacuum of ≈10^−3^ mbar, distance between Au source and sample of ≈25 cm).

### Characterization

XRD patterns of Cs_2_AgBiBr_6_ and the hybrid layer film were recorded on a diffractometer (PAN analytical EMPTREAN S3) equipped with Cu K*α* radiation source (*λ* = 1.5406 Å). The UV–vis spectra were measured with an ultraviolet‐visible spectrophotometer (TU‐1901). The surface morphology SEM images were recorded via a SEM (Zeiss Gemini 450) while the cross‐sectional image was obtained using a transmission electron microscope (TEM, JEM‐2100F). All the photonic synapse characteristics of the device were measured with a self‐constructed photoelectric test system, which included a semiconductor parameter analyzer (Keithley 2636B), a laser at 445 nm wavelength, TTL‐controlled optical shutters, and a dual‐channel arbitrary function generator (Tektronix AFG3022C). An optical power meter (Field Max *II*) was employed to obtain the power density of optical pulses. The polarization voltage was applied to the top Au electrode while the ITO bottom electrode was ground. All the characteristic tests were performed under ambient atmosphere and at room temperature.

### Face Recognition Method

The simulation for face recognition was built with MATLAB. Eventually, *β* was defined as 1000 A^−1^, and *η* equals to 10^−7^. Besides, the target value of the activation function $f_{i}^{\left(g\right)}$was 0.5 for the right class and −0.5 for other wrong classes during training process.

### Statistical Analysis

To evaluate the device variation, a waiting time of 5 min before each test were inputted to eliminate the influence of the previous stimulus. 2 optical pulses and 5 optical pulses were used to repeatedly stimuli the device 20 times, respectively. This variation test was performed on 28 devices. All beginnings of each stimulus were set to be the zero time and the value of EPSC at 1 s was taken for statistical analyses (Figure 3h). The blue and red dots shown in Figure 3h were the experiment results tested by our photonic devices, and the boxes contain values within the upper and lower quartiles (25%–75%), which reflect the central location and spread range of data distribution. Origin pro and Excel software were used for the analysis of the variation of 28 devices. The variation value (0.078 for 2 stimuli and 0.075 for 5 stimuli) of cycle to cycle was calculated by (max − min)/average).

## Conflict of Interest

The authors declare no conflict of interest.

## Supporting information

Supporting Information

## Data Availability

The data that support the findings of this study are available from the corresponding author upon reasonable request.

## References

[1] C.‐Y. Wang , S.‐J. Liang , S. Wang , P. Wang , Z. a. Li , Z. Wang , A. Gao , C. Pan , C. Liu , J. Liu , H. Yang , X. Liu , W. Song , C. Wang , B. Cheng , X. Wang , K. Chen , Z. Wang , K. Watanabe , T. Taniguchi , J. J. Yang , F. Miao , Sci. Adv. 2020, 6, eaba6173. 32637614 10.1126/sciadv.aba6173 PMC7314516

[2] C. Yang , Nature 2020, 579, 32.32132685

[3] L. Mennel , J. Symonowicz , S. Wachter , D. K. Polyushkin , A. J. Molina‐Mendoza , T. Mueller , Nature 2020, 579, 62. 32132692 10.1038/s41586-020-2038-x

[4] S. Manipatruni , D. E. Nikonov , I. A. J. N. P. Young , Nat. Phys. 2018, 14, 338.

[5] C. Liu , X. Yan , X. Song , S. Ding , D. W. Zhang , P. Zhou , Nat. Nanotechnol. 2018, 13, 404. 29632398 10.1038/s41565-018-0102-6

[6] C. M. Yang , T. C. Chen , D. Verma , L. J. Li , B. Liu , W. H. Chang , C. S. Lai , Adv. Funct. Mater. 2020, 30, 2001598.

[7] P. Yao , H. Wu , B. Gao , J. Tang , Q. Zhang , W. Zhang , J. J. Yang , H. Qian , Nature 2020, 577, 641. 31996818 10.1038/s41586-020-1942-4

[8] F. Cai , J. M. Correll , S. H. Lee , Y. Lim , V. Bothra , Z. Zhang , M. P. Flynn , W. D. Lu , Nat. Electron. 2019, 2, 290.

[9] F. Zhou , Z. Zhou , J. Chen , T. H. Choy , J. Wang , N. Zhang , Z. Lin , S. Yu , J. Kang , H.‐S. P. Wong , Nat. Nanotechnol. 2019, 14, 776. 31308498 10.1038/s41565-019-0501-3

[10] F. Zhou , Y. Chai , Nat. Electron. 2020, 3, 664.

[11] R. A. John , J. Acharya , C. Zhu , A. Surendran , S. K. Bose , A. Chaturvedi , N. Tiwari , Y. Gao , Y. He , K. K. Zhang , Nat. Commun. 2020, 11, 3211. 32587241 10.1038/s41467-020-16985-0 PMC7316775

[12] Q.‐B. Zhu , B. Li , D.‐D. Yang , C. Liu , S. Feng , M.‐L. Chen , Y. Sun , Y.‐N. Tian , X. Su , X.‐M. Wang , Nat. Commun. 2021, 12, 1798. 33741964 10.1038/s41467-021-22047-w PMC7979753

[13] J. Yu , X. Yang , G. Gao , Y. Xiong , Y. Wang , J. Han , Y. Chen , H. Zhang , Q. Sun , Z. L. Wang , Sci. Adv. 2021, 7, eabd9117. 33731346 10.1126/sciadv.abd9117 PMC7968845

[14] S. Wang , C.‐Y. Wang , P. Wang , C. Wang , Z.‐A. Li , C. Pan , Y. Dai , A. Gao , C. Liu , J. Liu , Natl. Sci. Rev. 2021, 8, nwaa172. 34691573 10.1093/nsr/nwaa172 PMC8288371

[15] T.‐Y. Wang , J.‐L. Meng , Q.‐X. Li , Z.‐Y. He , H. Zhu , L. Ji , Q.‐Q. Sun , L. Chen , D. W. Zhang , Nano Energy 2021, 89, 106291.

[16] G. Feng , J. Jiang , Y. Li , D. Xie , B. Tian , Q. Wan , Adv. Funct. Mater. 2021, 31, 2104327.

[17] L. Sun , Z. Wang , J. Jiang , Y. Kim , B. Joo , S. Zheng , S. Lee , W. J. Yu , B.‐S. Kong , H. Yang , Sci. Adv. 2021, 7, eabg1455. 33990331 10.1126/sciadv.abg1455 PMC8121431

[18] C. Du , F. Cai , M. A. Zidan , W. Ma , S. H. Lee , W. D. Lu , Nat. Commun. 2017, 8, 2204. 29259188 10.1038/s41467-017-02337-y PMC5736649

[19] R. Midya , Z. Wang , S. Asapu , X. Zhang , M. Rao , W. Song , Y. Zhuo , N. Upadhyay , Q. Xia , J. J. Yang , Adv. Intell. Syst. 2019, 1, 1900084.

[20] J. Moon , W. Ma , J. H. Shin , F. Cai , C. Du , S. H. Lee , W. D. Lu , Nat. Electron. 2019, 2, 480.

[21] Y. Zhong , J. Tang , X. Li , B. Gao , H. Qian , H. Wu , Nat. Commun. 2021, 12, 408. 33462233 10.1038/s41467-020-20692-1 PMC7814066

[22] J. W. Lee , D. H. Kim , H. S. Kim , S. W. Seo , S. M. Cho , N. G. Park , Adv. Energy Mater. 2015, 5, 1501310.

[23] B. Conings , J. Drijkoningen , N. Gauquelin , A. Babayigit , J. D'Haen , L. D'Olieslaeger , A. Ethirajan , J. Verbeeck , J. Manca , E. Mosconi , Adv. Energy Mater. 2015, 5, 1500477.

[24] W. Ning , F. Gao , Adv. Mater. 2019, 31, 1900326. 10.1002/adma.201900326 31025419

[25] G. Longo , S. Mahesh , L. R. Buizza , A. D. Wright , A. J. Ramadan , M. Abdi‐Jalebi , P. K. Nayak , L. M. Herz , H. J. Snaith , ACS Energy Lett. 2020, 5, 2200.

[26] X. Yang , Y. Chen , P. Liu , H. Xiang , W. Wang , R. Ran , W. Zhou , Z. Shao , Adv. Funct. Mater. 2020, 30, 2001557.

[27] B. Tian , J. Wang , S. Fusil , Y. Liu , X. Zhao , S. Sun , H. Shen , T. Lin , J. Sun , C. Duan , M. Bibes , A. Barthélémy , B. Dkhil , V. Garcia , X. Meng , J. Chu , Nat. Commun. 2016, 7, 11502. 27143121 10.1038/ncomms11502 PMC4857482

[28] G. Wu , B. Tian , L. Liu , W. Lv , S. Wu , X. Wang , Y. Chen , J. Li , Z. Wang , S. Wu , H. Shen , T. Lin , P. Zhou , Q. Liu , C. Duan , S. Zhang , X. Meng , S. Wu , W. Hu , X. Wang , J. Chu , J. Wang , Nat. Electron. 2020, 3, 43.

[29] B. Tian , L. Liu , M. Yan , J. Wang , Q. Zhao , N. Zhong , P. Xiang , L. Sun , H. Peng , H. Shen , T. Lin , B. Dkhil , X. Meng , J. Chu , C. Duan , Adv. Electron. Mater. 2019, 5, 1800600.

[30] M. Yan , Q. Zhu , S. Wang , Y. Ren , G. Feng , L. Liu , H. Peng , Y. He , J. Wang , P. Zhou , X. Meng , X. Tang , J. Chu , B. Dkhil , B. Tian , C. Duan , Adv. Electron. Mater. 2021, 7, 2001276.

[31] B. Tian , N. Zhong , C. Duan , Chin. Phys. B 2020, 29, 097701.

[32] C. Duan , W. R. Mei , J. Hardy , S. Ducharme , J. Choi , P. A. Dowben , Europhys. Lett. 2003, 61, 81.

[33] S. Kumar , I. Hassan , M. Regue , S. Gonzalez‐Carrero , E. Rattner , M. A. Isaacs , S. Eslava , J. Mater. Chem. A 2021, 9, 12179.

[34] J. Gong , H. Yu , X. Zhou , H. Wei , M. Ma , H. Han , S. Zhang , Y. Ni , Y. Li , W. Xu , Adv. Funct. Mater. 2020, 30, 2005413.

[35] Z.‐D. Luo , X. Xia , M.‐M. Yang , N. R. Wilson , A. Gruverman , M. Alexe , ACS Nano 2019, 14, 746. 10.1021/acsnano.9b07687 31887010

[36] P. Yao , H. Wu , B. Gao , S. B. Eryilmaz , X. Huang , W. Zhang , Q. Zhang , N. Deng , L. Shi , H.‐S. P. Wong , Nat. Commun. 2017, 8, 15199. 28497781 10.1038/ncomms15199 PMC5437298

[37] M. Prezioso , F. Merrikh‐Bayat , B. Hoskins , G. C. Adam , K. K. Likharev , D. B. Strukov , Nature 2015, 521, 61. 25951284 10.1038/nature14441

[38] F. Igbari , R. Wang , Z.‐K. Wang , X.‐J. Ma , Q. Wang , K.‐L. Wang , Y. Zhang , L.‐S. Liao , Y. Yang , Nano Lett. 2019, 19, 2066. 30803237 10.1021/acs.nanolett.9b00238

[39] J. Lao , W. Xu , C. Jiang , N. Zhong , B. Tian , H. Lin , C. Luo , J. Travas‐sejdic , H. Peng , C. Duan , J. Mater. Chem. C 2021, 9, 5706.

