# Supplementary material for: Ultralow‐Power Machine Vision with Self‐Powered Sensor Reservoir
Source: Adv Sci (Weinh). 2022 Mar 13;9(15):2106092. doi: 10.1002/advs.202106092 (PMC9130913; doi:10.1002/advs.202106092)
Supplement: Supplementary file 1 — Supporting Information [file ADVS-9-2106092-s001.pdf]

ADVANCED SCIENCE
Open Access

Supporting Information

for *Adv. Sci.*, DOI 10.1002/advs.202106092

Ultralow-Power Machine Vision with Self-Powered Sensor Reservoir

*Jie Lao, Mengge Yan, Bobo Tian*, Chunli Jiang, Chunhua Luo, Zhuozhuang Xie, Qiuxiang Zhu, Zhiqiang Bao, Ni Zhong, Xiaodong Tang, Linfeng Sun, Guangjian Wu, Jianlu Wang, Hui Peng*, Junhao Chu and Chungang Duan**

# Supporting Information

## Ultralow-power machine vision with self-powered sensor reservoir

*Jie Lao, Mengge Yan, Bobo Tian*, Chunli Jiang, Chunhua Luo, Zhuozhuang Xie, Qiuxiang Zhu, Zhiqiang Bao, Ni Zhong, Xiaodong Tang, Linfeng Sun, Guangjian Wu, Jianlu Wang, Hui Peng*, Junhao Chu and Chungang Duan**

**Supplementary Note 1:**

**The structural characterization of Au/P(VDF-TrFE)/$Cs_2AgBiBr_6$/ITO device**

The top-view scanning electron microscopy (SEM) images in Figure S1a-b indicate that the surface of $Cs_2AgBiBr_6$ is smooth and dense, and $Cs_2AgBiBr_6$/P(VDF-TrFE) hybrid layer also implys a well morphology. The cross-sectional SEM image of the two-terminal device (Figure S1c) gives that the thickness of P(VDF-TrFE) and $Cs_2AgBiBr_6$ was ~85 and 165 nm, respectively. X-ray diffraction (XRD) was performed on both $Cs_2AgBiBr_6$ single layer and $Cs_2AgBiBr_6$/P(VDF-TrFE) hybrid films (Figure S2). The XRD pattern of peaks at 13.7°, 15.9°, 22.5°, 26.5°, 27.5°, 31.9°, 35.7°, 39.4°, 45.7° are assigned to (111), (002), (022), (113), (222), (004), (024), (224) and (044) planes of the crystalline $Cs_2AgBiBr_6$, which could be well agreed with the octahedral crystal structure simulated by Woodwards et al.[1] The extra peak at 19.4° in $Cs_2AgBiBr_6$/P(VDF-TrFE) hybrid films was corresponding to P(VDF-TrFE) according to previous reports.[2, 3] The UV–vis absorption spectra in Figure S3 imply that $Cs_2AgBiBr_6$ have an absorption peak close to 445 nm, and the addition layer of P(VDF-TrFE) didn't affect its absorbance characteristic. The above-mentioned results firmly demonstrate that the $Cs_2AgBiBr_6$ perovskite film and $Cs_2AgBiBr_6$/P(VDF-TrFE) hybrid layer film were successfully synthesized and well combined.

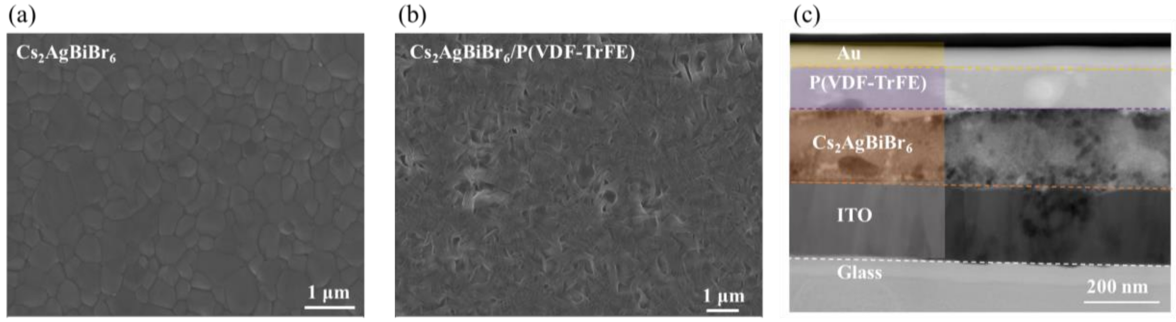


**Figure S1.** (a-b) Top view SEM image of $Cs_2AgBiBr_6$ film and $Cs_2AgBiBr_6$/P(VDF-TrFE) hybrid layer film. (c) The cross-sectional SEM image of the Au/P(VDF-TrFE)/ $Cs_2AgBiBr_6$/ITO device.

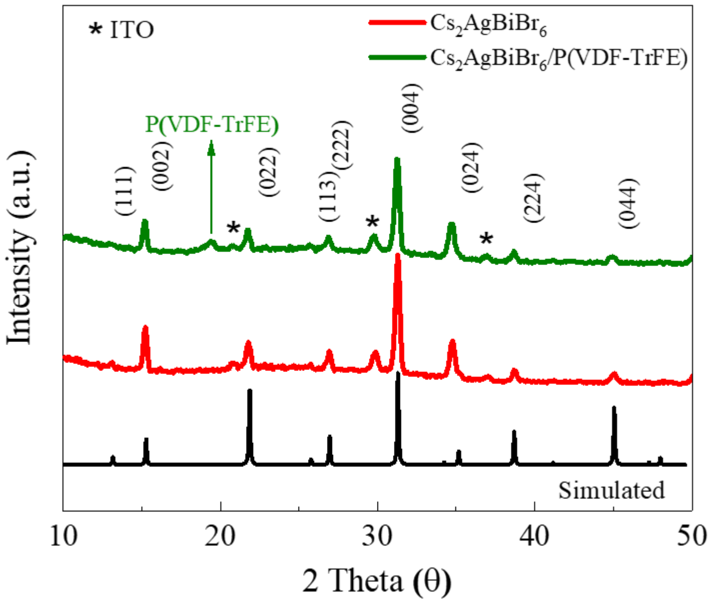


**Figure S2.** X-ray diffraction (XRD) pattern of both $Cs_2AgBiBr_6$ layer and $Cs_2AgBiBr_6$/P(VDF-TrFE) hybrid films.

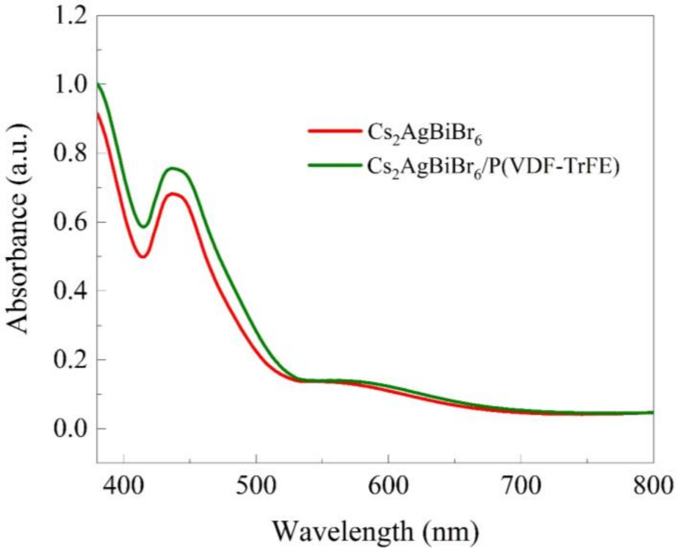


**Figure S3.** Absorbance of $Cs_2AgBiBr_6$ film and $Cs_2AgBiBr_6$/ P(VDF-TrFE) hybrid layer film.

**Supplementary note 2:**

**The photocurrents affected by polarization orientation**

In order to quantify how polarization of ferroelectric P(VDF-TrFE) layer affects $Cs_2AgBiBr_6$, the device was investigated by applying 10 optical pulses after upward polarization and downward polarization, and the pristine state was presented as comparison (Figure S4a-c). The pristine state corresponds to the state without any polarization at the ferroelectric layer (Note that all photonic-synaptic behaviors mentioned in this work were stimulated at pristine state). Upward polarization and downward polarization state refer to the polarization direction of P(VDF-TrFE) pointing up or down from the ITO substrate (inset of Figure S4a-c). The ferroelectricity of P(VDF-TrFE) is confirmed by the *P-V* hysteresis loop of an Au/P(VDF-TrFE)/ITO capacitor (Figure S5). The device was set into the upward/downward polarization state by applying electrical pulse with voltage of -10 V/+10 V before applying optical stimulation. After the P(VDF-TrFE) was polarized, the electrical pulse was removed, 10 optical pulses with power density of 36.6 $\mu W/cm^2$ and width of 100 ms were applied. The facilitation ($A_n/A_1$) stimulated in these three states mentioned above were carefully compared at each optical pulse in Figure 1h. Obviously, in the upward polarization, the EPSC facilitation was larger than the pristine state under the same illumination, and smaller than the pristine state while in downward polarization state. The reason for this phenomenon could be explained by the polarization of P(VDF-TrFE) ferroelectric layer, which would affect the band bending between the interface of P(VDF-TrFE) and $Cs_2AgBiBr_6$. The high binding energy of the valence electron states in P(VDF-TrFE) layer gives an energy potential well for hole carriers at the P(VDF-TrFE)/$Cs_2AgBiBr_6$ interface. This energy potential well makes the photon-generated hole carriers difficult to migrated to the Au electrode, which extends the lifetime of EPSC after removing the optical stimulus. This is the reason of the observed long-tail photocurrents and their facilitation under multi optical

stimuluses. In the upward polarization, energy potential well is deeper, which gives a photocurrent with smaller amplitude but longer tail under each optical pulse. Their longer tails enhance the facilitation effect (coupling strength) under multi optical stimuluses (Figure S4e). In the downward polarization, energy potential well is reduced, which gives a photocurrent with large amplitude but short tail under each optical pulse. Their short tails weaken the facilitation effect (coupling strength) under multi optical stimuluses (Figure S4f).

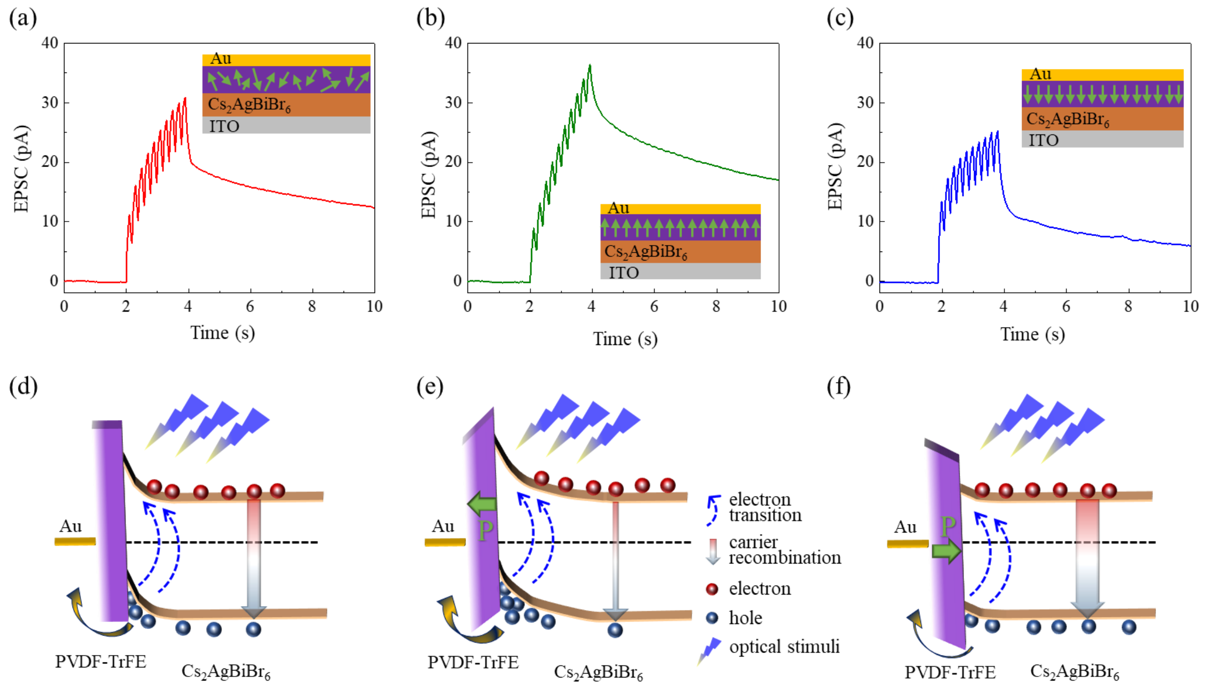


**Figure S4.** (a-c) EPSC triggered by 10 optical pulses in pristine state, upward and downward polarization states at Au/P(VDF-TrFE)/$Cs_2AgBiBr_6$/ITO device. The power density and width of the optical pulses are 36.6 μW/cm$^2$ and 100 ms, respectively. All measurements were performed with 0 V bias. (d-f) Suggested band diagram of the Au/P(VDF-TrFE)/$Cs_2AgBiBr_6$/ITO device under different polarization orientation.

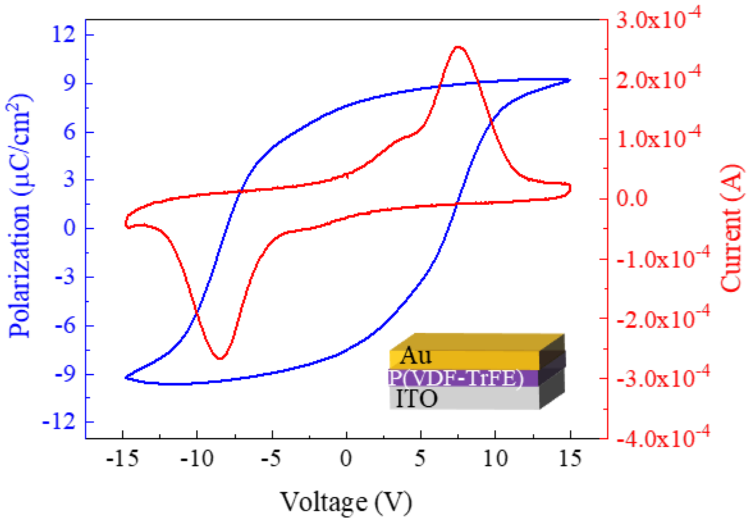


**Figure S5.** *P-V* hysteresis loop of an Au/Cr/ P(VDF-TrFE)/ITO capacitor.

**Supplementary note 3:**

**The optical artificial synapse characterization of Au/P(VDF-TrFE)/$Cs_2AgBiBr_6$/ITO device**

Figure S5 shows the excitatory post-synaptic current (EPSC) of the Au/P(VDF-TrFE)/$Cs_2AgBiBr_6$/ITO device. The EPSC was induced by an optical pulse with width of 100 ms and power density of 36.6 μW/cm$^2$. It is clear that the EPSC abruptly reaches to ~10.6 pA during the optical stimulation and then gradually decreased to the initial level. It is resembled to the change of EPSC in a biological synapse. The EPSC can be modulated by the width and power density of the optical pulse (Figure S6a-d). Figure S6a shows the EPSC increased with the optical pulse width with fixed power density of 36.6 μW/cm$^2$. When the pulse width increases from 0.1 s to 4.5 s, the EPSC increases from 10.9 to 145.3 pA, achieving 1233% increase. The EPSC could also be modulated by the increase of the power density of optical pulse when the pulse width was fixed (100 ms), with the EPSC increased 524% (from 10.1 to 63.1 pA) (Figure S6c-d).

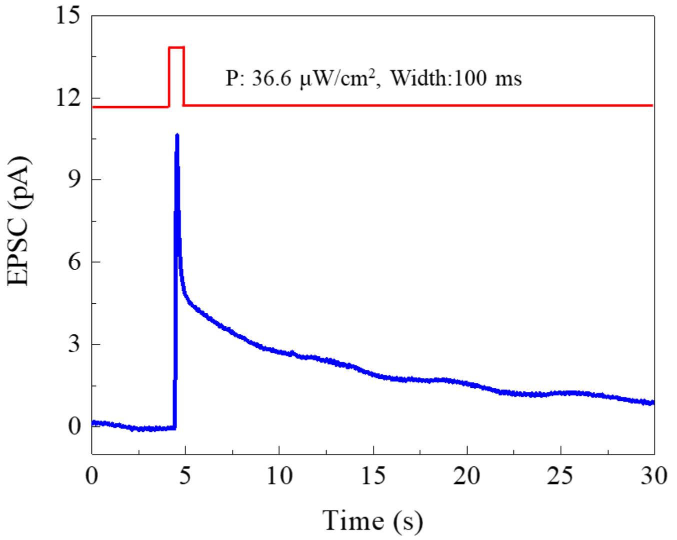


**Figure S6.** EPSC triggered by single optical pulse with power density of 36.6 μW/cm$^2$ and pulse width of 100 ms.

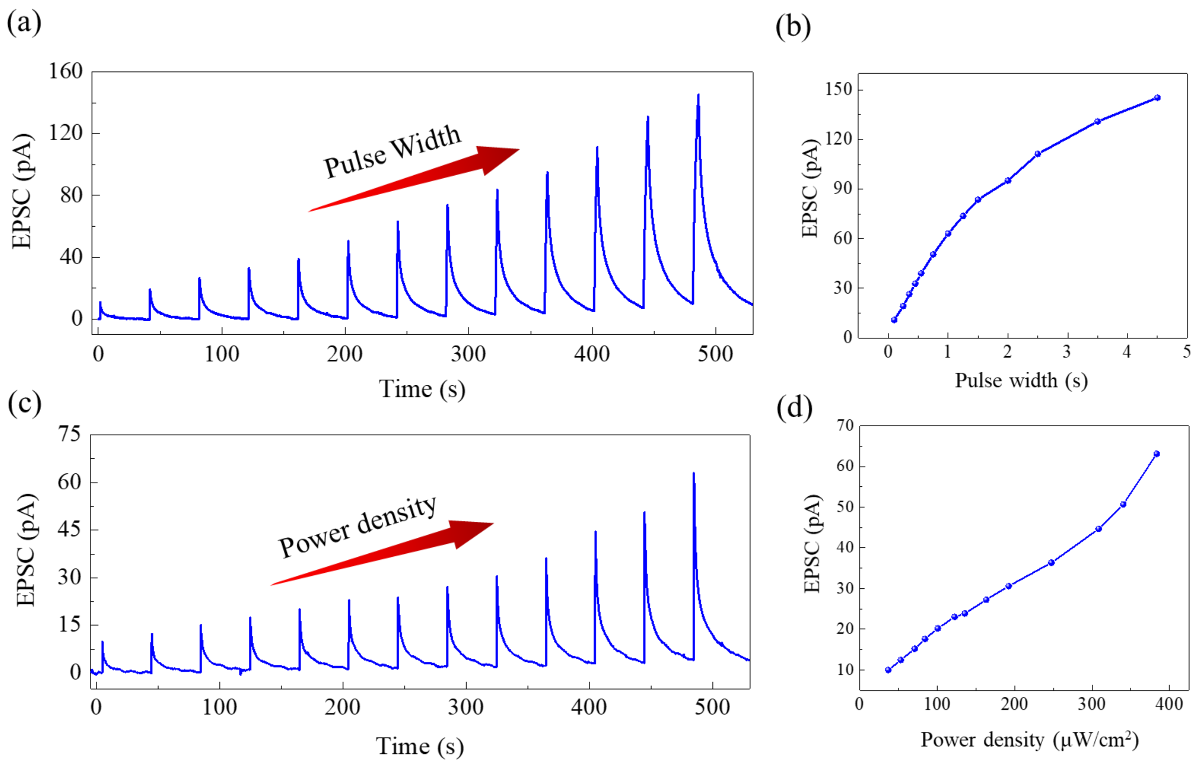


**Figure S7.** (a) EPSC triggered by different width of optical pulse with fixed power density (36.6 μW/cm$^2$). (b) Dependence of the EPSC and the pulse width. (c) EPSC triggered by single optical pulse with different power density. (d) Dependence of the EPSC triggered by an optical pulse on the power density (The pulse width was fixed on 100 ms).

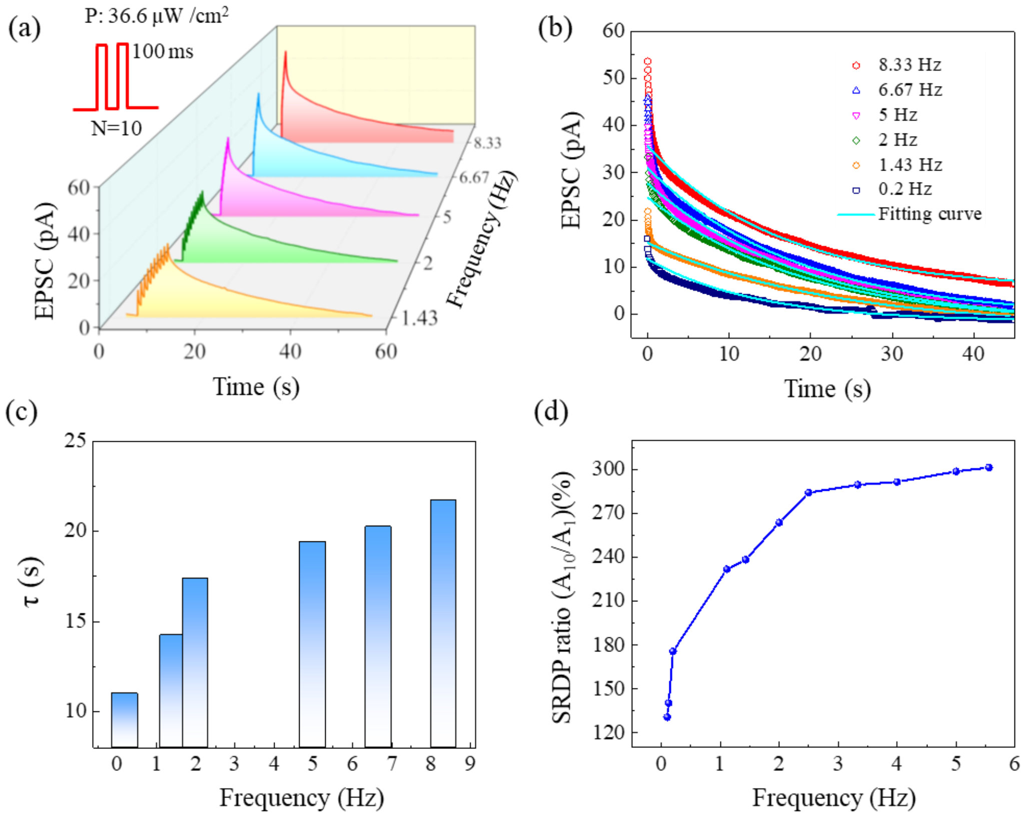


**Figure S8.** (a) SRDP triggered by different frequency of ten optical pulses. (b-c) Decay curves (b) and corresponding decay time (τ) (c) of SRDP in (a). Frequency dependence of SRDP ratio (defined as $A_{10}/A_1$) of the $Cs_2AgBiBr_6$-based photonic device.

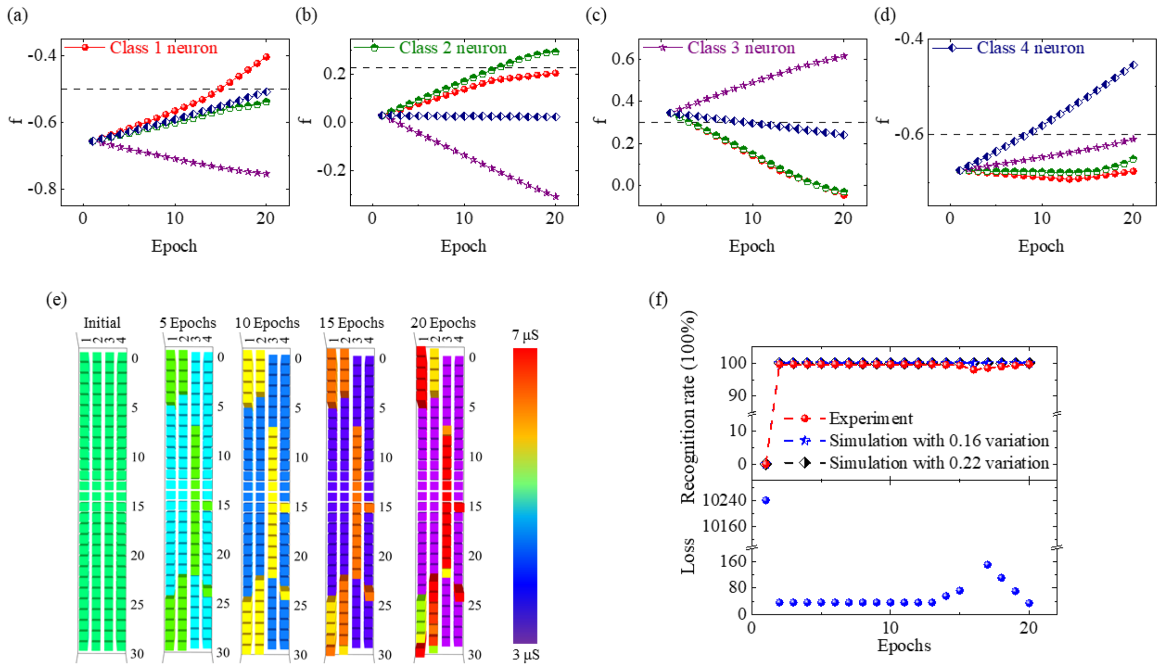


**Figure S9.** Training a readout memristor network with uniform initial weight. (a-d) The evolution of readout function (f) of the corresponding targeted neuron with training epochs. (e) Weights of memristors in 28×4 array are updated during each training epoch. (f) Top panel: the recognition rate for classification of class 1. Both experimental *y(t)* spaces and simulated *y(t)* spaces with a random variation of 0.16 and 0.22 are involved to obtain the recognition rate. Bottom panel: The failed number for classification of class 1 in 10240 tests.

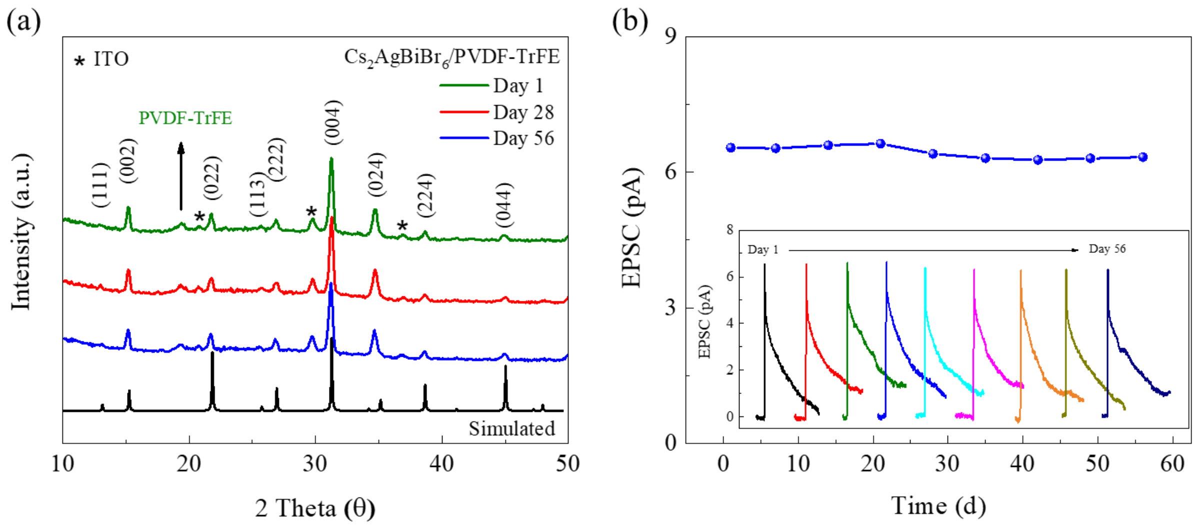


**Figure S10.** Stability of $Cs_2AgBiBr_6$-based photonic device. (a) XRD patterns of $Cs_2AgBiBr_6$/P(VDF-TrFE) hybrid film before and after eight week's storage in ambient environment. (b) EPSC triggered by a single optical pulse (power density and pulse width was 28.3 μW/cm$^2$ and 100 ms, respectively) before and after eight week's storage (Inset showed the EPSC tested from day 1 to day 56).

**Supplementary note 4**

During the In-sensor reservoir computing for image classification, the amplitude of EPSC at 35 s are collected from 28 reservoir devices as the new feature space *y(t)*. An activation function was used to further differentiate the intensity distribution in *y(t)* and convert *y(t)* to voltage inputs for the following network. The activation function is shown below:

$V_j(n)$ = tanh(6×($V_j'(n)$ / $\sum_{j=1}^{25} V_j'(n)$/max($V_j'$)) -3) (1)

with

$V'_j = I_j/\max(I_i, i = 1, 2, \ldots, 28)$ (2)

Here $I_j$ is the EPSC at 35 s, and $V_j(n)$ is the voltage that used as the input in the simulation for the *n*th image.

Following the flow chart in the right panel of Figure 4c, a training simulation for image classification are performed on the following 28×4 memristor network using the new *y(t)* inputs and Manhattan update rule.[4, 5] $V_j(n)$ is the input voltage representing the column j of *n*th image, $W_{ij}$ is the conductance of each memristor in the simulation arrays, β is a parameter that controls the non-linearity, $f_i^{(g)}$(n) is the target value of the *i*th image and η is a constant scaling the training rate. During the training process, each class was inputted three times repeatedly. The measured EPSC at t = 35 s of each class were processed via equation (1) and then applied to the array. Initially, all weights of memristors in array are programmed to same state of 5 μS. The array weights and readout function are updated with the training epoch. Obviously, the readout function value of array separates each other and the targeted neuron has highest readout value as training epoch number increases. The readout of targeted neuron exceeds the threshold value 0.1

**References**

[1] E.T. McClure, M.R. Ball, W. Windl, P.M. Woodward, *Chem. Mater.* **2016**, *28*, 1348-1354.
[2] E. Jia, D. Wei, P. Cui, J. Ji, H. Huang, H. Jiang, S. Dou, M. Li, C. Zhou, W. Wang, *Adv. Sci.* **2019**, *6*, 1900252.
[3] B. Tian, X. Bai, Y. Liu, P. Gemeiner, X. Zhao, B. Liu, Y. Zou, X. Wang, H. Huang, J. Wang, *Appl. Phys. Lett.* **2015**, *106*, 092902.
[4] P. Yao, H. Wu, B. Gao, S.B. Eryilmaz, X. Huang, W. Zhang, Q. Zhang, N. Deng, L. Shi, H.-S.P. Wong, *Nat. Commun.* **2017**, *8*, 1-8.
[5] M. Prezioso, F. Merrikh-Bayat, B. Hoskins, G.C. Adam, K.K. Likharev, D.B. Strukov, *Nature* **2015**, *521*, 61-64.
